# Electrospun Porous Nanofibers: Pore−Forming Mechanisms and Applications for Photocatalytic Degradation of Organic Pollutants in Wastewater

**DOI:** 10.3390/polym14193990

**Published:** 2022-09-23

**Authors:** Xianyang Cao, Wei Chen, Ping Zhao, Yaoyao Yang, Deng-Guang Yu

**Affiliations:** School of Materials and Chemistry, University of Shanghai for Science and Technology, 516 Jungong Road, Shanghai 200093, China

**Keywords:** electrospinning, nanofiber, porous structure, pore−forming mechanism, photocatalysis

## Abstract

Electrospun porous nanofibers have large specific surface areas and abundant active centers, which can effectively improve the properties of nanofibers. In the field of photocatalysis, electrospun porous nanofibers can increase the contact area of loaded photocatalytic particles with light, shorten the electron transfer path, and improve photocatalytic activity. In this paper, the main pore−forming mechanisms of electrospun porous nanofiber are summarized as breath figures, phase separation (vapor−induced phase separation, non−solvent−induced phase separation, and thermally induced phase separation) and post−processing (selective removal). Then, the application of electrospun porous nanofiber loading photocatalytic particles in the degradation of pollutants (such as organic, inorganic, and bacteria) in water is introduced, and its future development prospected. Although porous structures are beneficial in improving the photocatalytic performance of nanofibers, they reduce their mechanical properties. Therefore, strategies for improving the mechanical properties of electrospun porous nanofibers are also briefly discussed.

## 1. Introduction

As a “top−down” membrane preparation technology, electrospinning technology can produce continuous fibers with diameters ranging from microns to nanometers [1]. It also has the advantages of easy operation, low cost, high efficiency, high flexibility, a large number of fiber membranes within a short time, easy control of the fiber diameter and orientation, and easy control of the fiber microstructure [2,3,4] (porous [5], nano−protrusions [6], folds [7], branches [8], hollow [9], core−sheath [10], ribbons [11], and beads−on−the−string [12]). Nanofibers prepared by electrospinning usually have good biocompatibility and degradability and can carry drugs into the human body. Nanofibers have small size effect, good fiber homogeneity, large specific surface area and interfiber porosity. Electrospinning [13,14,15,16] has been continuously upgraded and optimized with in−depth study of their working processes. These include coaxial electrospinning [17,18], side−by−side electrospinning [19,20], tri−axial electrospinning [21,22], tri−fluid side−by−side electrospinning [23], and other multiple−fluid electrospinning [24] that have been developed, providing a strong guarantee for the preparation of complex structured nanofibers.

The process and technical principle of electrospinning is driven by the propelling pump while the spinning solution is extruded from the spinneret of the syringe. When no external electric field force is applied, a spherical droplet is formed under the action of surface tension and gravity. When an electric field force is applied, the droplet is connected to a high−voltage power source, and its surface is instantly covered by the same charge. The repulsive force and surface tension between these charges cancel each other out, and the droplet becomes unstable. If the repulsive force is strong enough to overcome the surface tension, the droplet will become conical (Taylor cone) and jet down from the spinneret. As the diameter of the jet continues to decrease until it bends, the solvent in the polymer jet evaporates rapidly in a short period of time, and finally solidifies into fibers with diameters ranging from nanometers to micrometers. The structure and morphology of the prepared fibers are affected by three sets of parameters: (1) system parameters (such as solution viscosity, polymer molecular weight, polymer solution concentration, electrical conductivity, dielectric constant, and surface tension); (2) process parameters (such as the magnitude of the applied voltage, the distance from the tip of the spinneret to the collection device, the flow rate of the spinning precursor solution); and (3) Environmental parameters (such as ambient temperature and humidity). These parameters will have a certain impact on the final morphology of the fiber. For example, increasing the voltage within a certain range will increase the charge density on the jet surface and increase the repulsive force, thus forming fibers with smaller diameters. However, if the voltage is too high, the formation of the Taylor cone will be hindered, resulting in the inability to spin normally. If the voltage and polymer solution concentration are too low, this may result in the production of some beads, which do not form continuous fibers of uniform diameter. If the voltage and polymer concentration are too high, the spinning process will be difficult, and the selection of appropriate experimental parameters can electrospin continuous, smooth, and uniform nanofibers.

**Figure 1:**
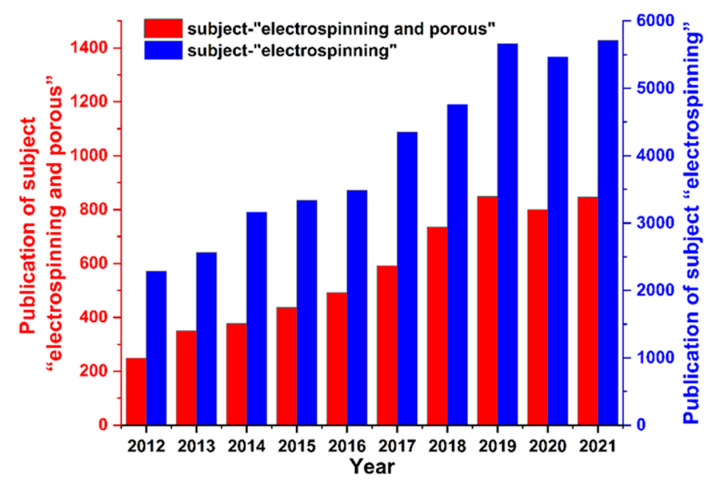
Research status of electrospinning porous nanofibers.

The porous structure is beneficial to the increase of the specific surface area of fibers, increase of ion transport channels, shortening ion transport paths, and enrichment of the active sites on the fiber surface and the internal space [25,26,27]. This results in electrospun porous nanofibers with a wide range of application prospects in the fields of photocatalysis [28], sensors [29], capacitors [30], drug delivery [31], and filtration [32]. As shown in Figure 1, research over the past ten years was searched using the “electrospinning” and “electrospinning and porous” keywords. The results show that the amoun of related research on electrospun porous nanofibers increased annually, and the proportion of studies related to electrospinning remained stable.

Photocatalysis was discovered in 1967 when Akira Fujishima decomposed water into oxygen and hydrogen through titanium dioxide single crystals under light [33]. It is a cleaning technology that continuously and efficiently degrades pollutants without causing secondary pollution. The principle of this technology is to use light to excite the photocatalyst. When light with energy greater than or equal to the energy gap is irradiated onto the photocatalytic particle, the electrons in its valence band (VB) are excited to jump to the conduction band (CB) [34], leading to the formation of corresponding holes in the VB, and electron–hole pairs are then easy to form due to the strong mutual attraction between electrons and holes. At this time, the two are separated from each other by the secondary excitation of light and diffused to the surface of the particles, producing a strong redox reaction with the pollutants [35]. The holes in the VB are good oxidants, and the electrons in the CB are good reducing agents. They generally react with H_2_O and O_2_ to generate highly oxidizing hydroxyl radicals (OH) and superoxide anions (O^2−^), which can directly oxidize organic matter into inorganic small molecules such as CO_2_ and H_2_O, without producing intermediate products, which is relatively clean and fast. During photocatalysis, the photocatalyst itself does not participate in the reaction but can accelerate the chemical reaction rate by promoting the separation of electron–hole pairs under the excitation of photons, thus improving the photocatalytic efficiency [36]. However, in the photocatalytic process, the loss of photocatalytic particles during use can lead to poor reusability. At this time, combining photocatalytic technology with the electrostatic spinning of porous fibers can not only improve photocatalytic efficiency but also avoid losses and improve reusability [37,38]. In this paper, we summarize the electrospinning mechanism for the preparation of porous nanofiber membranes, its application in photocatalysis, and the relationship between porous structure and the properties of nanofibers.

## 2. Pore−Forming Mechanism of Electrospun Porous Nanofibers

In the electrospinning process, some solvents with high volatility (dimethylformamide DCM [39,40,41,42], chloroform CHL [43,44,45,46], and tetrahydrofuran THF [47]) or solvents and polymers that are easy to remove in post−treatment (polyethylene oxide PEO [48,49,50] polyvinylpyrrolidone PVP [51,52]) are commonly used to form porous nanofibers. Solvents with high volatility can volatilize within a very short electrospinning time and form a porous structure [53]. Selective removal of solvents by post−treatment can also leave pores on the surface and inside [54,55]. The results show that the pore−forming mechanisms of electrospun porous nanofibers mainly include breath figures (BFs) [56,57,58], vapor induced phase separation (VIPS) [59,60], non−solvent induced phase separation (NIPS) [61,62], thermally induced phase separation (TIPS) [63,64], and selective removal (SR) [65,66].

### 2.1. Breath Figures (BFs)

**Figure 2:**
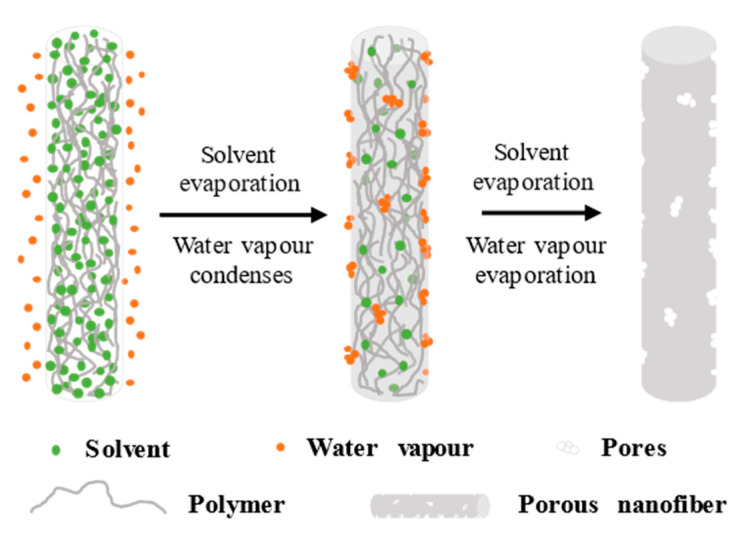
Schematic of the pore−forming mechanism of BFs.

This pore−forming mechanism is generally applicable to highly volatile solvents (DCM, CHL) and high environmental humidity. As shown in Figure 2, due to the rapid volatilization of the solvent during the electrospinning process, the heat on the surface of the fiber is taken away, significantly reducing the surface temperature. Then, the water vapor becomes extremely small droplets attached to the fiber when cooled, occupying space on the fiber surface. Finally, the complete evaporation of the solvent and the water vapor leaves circular pores on the fiber surface [67,68,69]. Relative humidity is an important factor in the pore−forming mechanism of BFs. When in high ambient humidity, the pore size range of this circular pore is generally 20−200 nm, and the porosity is more than 90%. However, as the ambient humidity decreases, the pore size becomes smaller and the porosity decreases. When the ambient humidity is low enough, the porous structure cannot be formed through this mechanism [47,56,70].

The circular holes are mainly formed in the later stage of the electrospinning process. Otherwise, these holes will be pulled into elliptical holes due to the electrostatic force. This phenomenon usually occurs when highly volatile solvents and hydrophobic polymers (polystyrene PS [71,72], polymethylmethacrylate PMMA [73], polylactic acid PLA [74,75]) are combined. If hydrophilic polymers, such as polyvinyl alcohol (PVA) and polyethylene oxide (PEO), are selected, then the surface will display folds rather than holes. For example, Megelski et al. [68] used CHL as a solvent and prepared hydrophobic PS and PMMA fibers with a certain porosity on the surface; consequently, the surface of the prepared hydrophilic PEO fibers displayed folds instead of pores. This phenomenon may be attributed to the rapid evaporation of the highly evaporative solvent during the spinning process and the condensation of the water vapor on the fiber surface to form droplets. If the polymer is hydrophobic, then the droplets will condense on the fiber surface and leave round holes after evaporation; if the polymer is hydrophilic, then the droplets will mix with the hydrophilic polymer, and even if the water vapor evaporates, the fiber surface will exhibit a pleated structure instead of a hole structure due to the interaction between the water vapor and the fiber.

The BFs pore−forming mechanism is simple and convenient, using a highly evaporative solvent to form pores in one step during the spinning process, but such pores only exist on the surface of the fiber, limiting the application.

### 2.2. Vapor Induced Phase Separation (VIPS)

**Figure 3:**
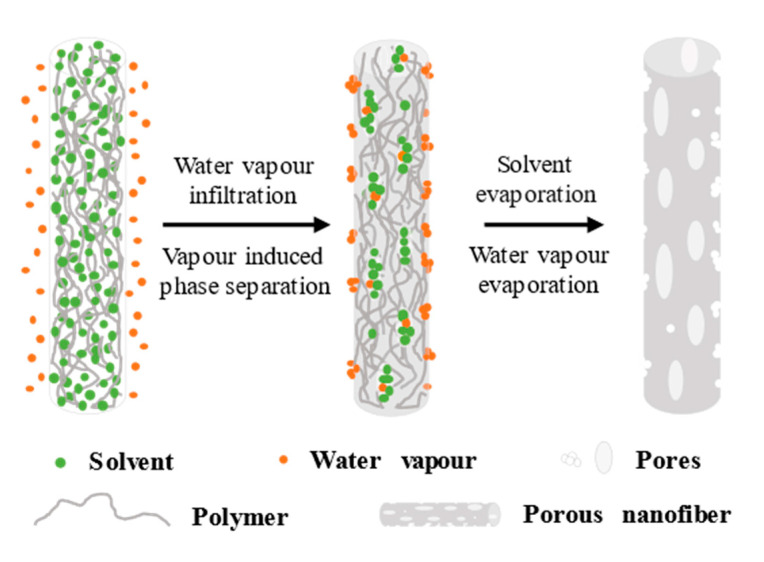
Schematic of the pore−forming mechanism of VIPS.

Unlike BFs, which require a highly evaporative solvent and poor water miscibility, VIPS requires a solvent with low evaporation and good water miscibility. As shown in Figure 3, when the polymer jet is stretched at high speed in a high−voltage electrostatic field, the water molecules in the air can easily penetrate the fiber and quickly mix with the solvent, acting as a non−solvent for the polymer, and resulting in the phase separation between the polymer and the solvent within a short time to form solvent−rich zones and polymer−rich zones. Finally, the polymer enrichment gradually solidifies into the fiber skeleton, while the solvent−rich region leaves pores inside the fiber due to solvent volatilization [47,69,76]. The pore formation of the BFs occurs after the polymer jet is stretched, so the shape of the pores is round and easy to distinguish. While the pores formed by VIPS are generally elliptical, the water molecules in the air mix quickly with the solvent. After the stretching of the electric field and complete volatilization, elliptical pores will be formed on the surface and inside of the fiber. The pore size of these elliptical pores is generally in the range of 50−300 nm, and the porosity can reach 92% [56,77,78].

Under the VIPS mechanism, dimethylformamide (DMF), dimethylacetamide (DMAC), and dimethyl sulfoxide (DMSO) are suitable solvents for pore formation. For example, Huang et al. [69] prepared PLA nanofibers with porous surfaces and interiors using DMSO as a single solvent. Given the low evaporation of DMSO, the water molecules had sufficient time to enter the interior of the fibers before the polymer was completely dried, thus inducing phase separation and creating pores inside. Lu et al. [76] discussed the influence of the volatility of different solvents and their miscibility with water on pore formation. Using a certain proportion of DMF with low volatility and good miscibility with water, the PS electrospun fiber surface displays pores under a certain humidity. If high volatile THF is selected, the vapor pressure is much higher than that of water despite the good miscibility of THF with water. Under the action of high concentration THF, the interface between the fiber and air is always in the state of THF saturation, preventing the entry and phase separation of water molecules. However, water molecules will condense on the fiber surface due to the volatilization of THF, and only pores will remain on the surface of the PS fiber after volatilization. If the appropriate proportions of THF and DMF are selected, pores will remain on the surface and inside the fiber.

The phase separation caused by the increase in polymer concentration during evaporative cooling and jet drying is a key step in the formation of pores in composite nanofiber sheaths, and if the evaporative drying process of the solvent is slow, then the phase separation process will decelerate, which will reduce the pore distribution density. In coaxial electrospinning, the core and sheath liquid flow rates are different, which affects the pore distribution density. For example, the core feed rate is high, slowing the evaporation of the solution jet and the polymer drying and reducing the pore density.

Unlike BFs, which can only form pores on the surface of fibers, VIPS can form pores on the surface of and inside the fibers, and both require only one solvent.

### 2.3. Non−Solvent Induced Phase Separation (NIPS)

NIPS requires a highly evaporative good solvent and a low evaporative non−solvent, which is miscible with the good solvent but cannot dissolve the polymer or can only dissolve a very small amount of the polymer; the non−solvent has a high boiling point and the dielectric constant for the good solvent and the choice of polymer is wide.

**Figure 4:**
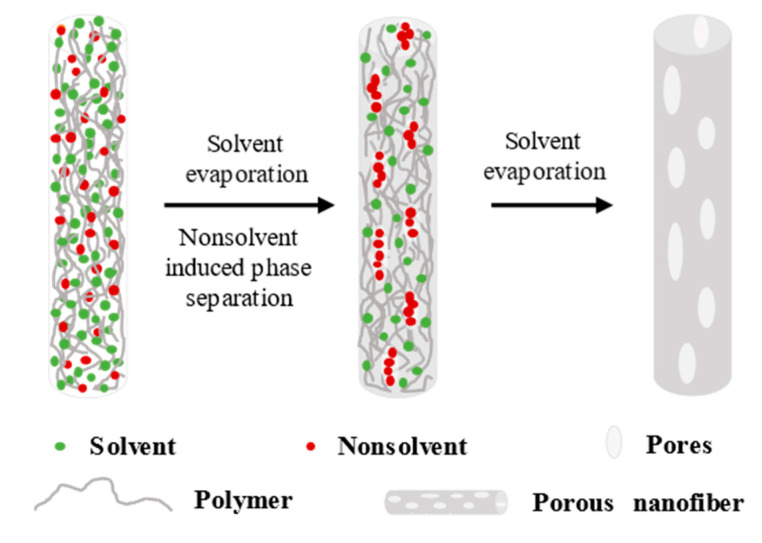
Schematic of the pore−forming mechanism of NIPS.

A certain amount of non−solvent to form a polymer/solvent/non−solvent ternary solution must be added to form pores in NIPS. The choice of solvent/non−solvent combination is very important. To achieve phase separation, the volatile order of solvent and non−solvent should be different: high volatility of good solvent and low volatility of non−solvent. As shown in Figure 4, the pore−forming mechanism of NIPS is a two−solvent system formed by a good solvent and a non−solvent. When the evaporation rate of the good solvent is higher than that of the non−solvent, the relative content of the good solvent will continuously decrease with the rapid evaporation of the good solvent, while the relative content of the non−solvent will increase, and when the good solvent evaporates completely, the original single−phase homogeneous ternary solution will become a two−phase heterogeneous binary solution within a very short time with only the non−solvent and the polymer. The non−solvent volatilization rate is slow, causing the non−solvent to remain in the polymer system, and pores are left at the location when the non−solvent volatilizes slowly and completely [69]. However, if the initial non−solvent content is extremely high, then the porous structure of the fiber surface transforms into a folded structure. The shapes of the pores formed by NIPS are similar to those of VIPS, both of which are pores formed when the polymer jet is stretched, and both are elliptical pores. The pores formed by NIPS are independent of external humidity, so the elliptical pore size is generally small, the range is generally 20−100 nm, with a minimum pore size of 3 nm. However, its porosity is low, less than 80% [61,79,80]. The preparation of nanofibers with porous structures on the surface and inside using a one−step method is difficult and requires the selection of a suitable solvent to non−solvent ratio and polymer content.

For example, Qi et al. [81] used a PLA/DCM/DMF ternary solution system to form a highly porous electrospun nanofiber. DCM is a good solvent, whereas DMF is a poor solvent with a very high molecular weight, and the evaporation rate of DCM is much higher than that of DMF during the electrospinning process. Moreover, the solution composition of the polymer passes through the phase boundary into the two−phase region within a short time due to the rapid evaporation of DCM. The liquid−liquid phase separation results in polymer−rich regions and solvent−rich regions and finally causes the solvent volatilization to form a hole. However, if the difference between the volatilization rates of the solvent and the non−solvent is insufficiently large, then the entry of the solution to the two−phase region will be very slow, eventually making the formation of pores difficult. Other two−solvent systems are used for porous nanofibers, such as acetone Ac/DMF [82], DMF/H_2_O [83], and DCM/hexane [84,85].

Yu et al. [86] used electrospinning to prepare porous polyacrylonitrile (PAN) fibers in one step by using the PAN/DMF/H_2_O ternary system at room temperature. The composition of the PAN solution approaches the equilibrium point of the ternary system as the non−solvent H_2_O content increases, indicating that strong phase separation may occur and result in nanofibers with porous structures on the surface and inside during electrospinning.

Thuy et al. [87] prepared composite nanofibers with a porous core−sheath structure and a hydrophobic surface using salicylic acid (SA) and polyethylene glycol (PEG) as the core material, PLA as the sheath material, and DCM and dimethylacetamide DMAC (9:1) as the solvent. PLA has excellent biodegradability and biocompatibility, can support cell attachment and proliferation, and has great application potential in tissue engineering and wound dressing. Core–sheath composite nanofibers can protect the core active agent from the environment and maintain its activity, and the porous structure is conducive to drug release, which can be controlled by modulating the porous core−sheath structure to achieve the desired release rate.

Song et al. [88] prepared a porous PLA filter membrane with a large specific surface area, a high filtration efficiency and a low pressure drop for aerosol particles less than 100 nm. This membrane can be used in filtration devices in harsh environments, such as aerospace and the ocean. The PLA porous filter membrane formation can be divided into three stages. In the first stage, the liquid phase system is homogeneous without any phase separation. Then, when the precursor solution is jetted down from the spinneret, the easy−to−volatilize DCM volatilizes first, while the difficult−to−volatilize DMF is left in the polymer system, at which point NIPS and BF occur simultaneously. Finally, the non−solvent evaporates, leaving only the dry polymer phase. The space occupied by the non−solvent phase becomes a pore space and is distributed inside the fiber.

Unlike BFs and VIPS, which require only one solvent for the fiber to have a porous structure, non−solvent induced separation usually requires two or more solvents, and the selection of solvents is also different from that of the former two.

### 2.4. Thermally Induced Phase Separation (TIPS)

**Figure 5:**
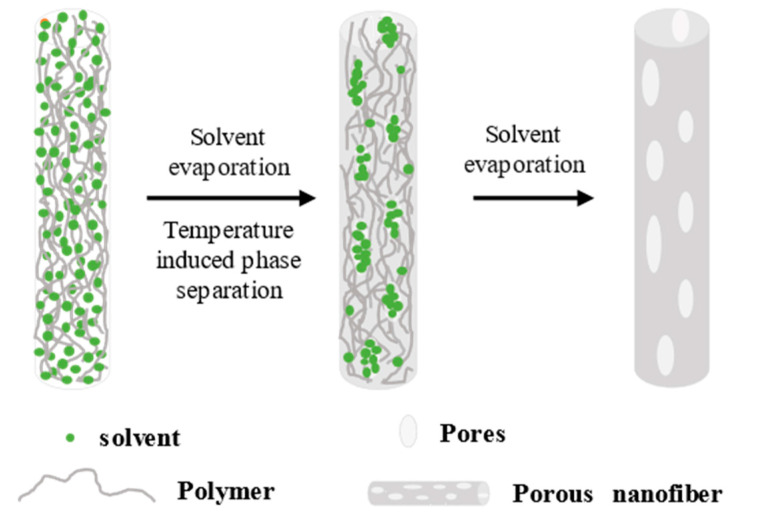
Schematic of the pore−forming mechanism of TIPS.

The pore−forming mechanism of TIPS is shown in Figure 5. (1) The polymer is dissolved in high−boiling−point and low−volatility solvents to form a homogeneous solution. (2) The precursor solution is electrospun into fibers. (3) Fiber cooling separates the polymer and nonvolatile solvent in the fiber, and the temperature difference is the driving force [89,90]. (4) The non−volatile solvent in the electrospun fiber is removed to form a porous structure. Due to the complicated conditions of the whole process, the formed pores have different shapes, with a small pore size generally in the range of 2−50 nm, and with a lower the porosity than the previous pore−forming mechanisms [64,91].

McCann et al. [91] electrospun fibers and fed them directly into a liquid nitrogen bath, obtaining porous structures after the solvent was removed in a vacuum. In contrast, the control group of nanofibers without liquid nitrogen treatment only exhibited surface texture roughness, indicating that TIPS was the driving force behind the formation of porous fibers. Previous research indicates that appropriate freezing temperature and sufficient solvent residue are the necessary conditions for the formation of a porous structure, so TIPS is only suitable for polymer solutions with low−vapor−pressure and high−boiling point solvents to ensure that the solvent will not evaporate rapidly during the spinning process and that a certain amount of residual solvent remains inside the fiber until the collection plate at low temperature is reached. Ye et al. [90] prepared porous polypropylene fibers by combining electrospinning and thermally induced phase separation. A certain amount of diluent was added to the electrospinning precursor and electrospun under a high temperature coil at 300 °C. The fibers were cooled, leading to phase separation. Finally, the diluent was extracted and removed, and pores appeared on the surface of and inside the fibers. The porous structure was also prepared by electrospinning and the TIPS of polyvinylidene fluoride PVDF, confirming the universal applicability of TIPS. Li et al. [89] prepared polycaprolactone (PCL) porous fibers by using a collector at −3.6 °C. The pores were mainly divided into two types, namely, a circular pore and a shallow polygonal concave surface on the fiber surface. The diameter distribution of the fibers with two different structures is similar, and the average diameters of the pores and the concave surfaces are similar because as the jet reaches the frozen collector, the solvent is freeze−dried before it is completely volatilized and removed after producing pores. At the freezing temperature of −3.6 °C, the moisture in the air is deposited and frozen on the collector plate, and the interaction of the residual solvent with the moisture in the environment may cause the formation of polygonal concave surfaces on the fiber surface. However, the electrospinning process is extremely short, and not all of the solvent interacts with the moisture in the environment before freeze−drying, creating pores and polygonal craters on the fiber surface.

Thus, the porous fiber membranes prepared by TIPS require harsh conditions and are unsuitable for industrial mass production.

### 2.5. Selective Removal (SR)

**Figure 6:**
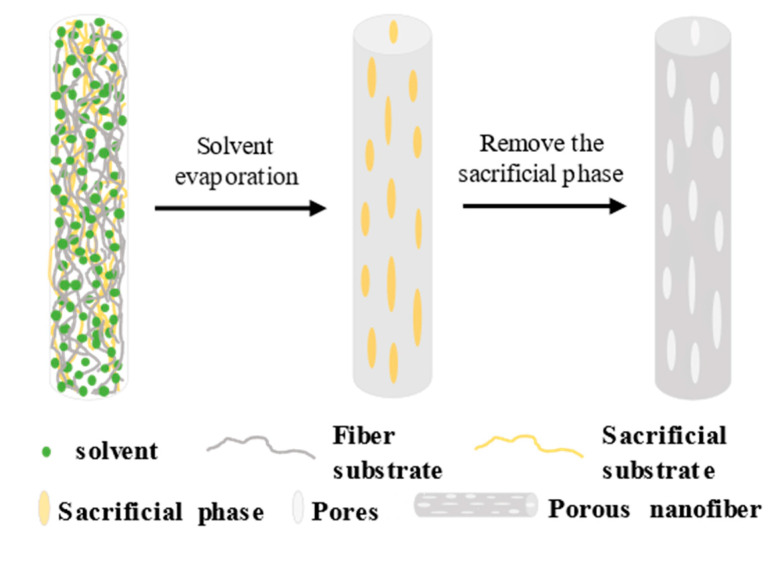
Schematic of the pore−forming mechanism of SR.

Unlike the above pore induction mechanisms, SR is a two−step process requiring post−treatment, as shown in Figure 6. The first step is the electrospinning of the fiber, and the second step is removing the sacrificial component, which requires careful control of the conditions under which the sacrificial phase (in the SR pore−forming mechanism, a certain polymer needs to be removed to prepare a porous structure. For example, Xu uses PAN and PEG as spinning polymers, as well as a sacrificial phase−PEG, thereby preparing porous PAN nanofibers [91]) is removed to maintain the integrity of the fiber backbone [92,93]. Most of the sacrificial components can be removed from the fiber by appropriate extraction or heat treatment, but a small amount of the sacrificial phase may remain in the polymer matrix and affect the performance of the fiber. The pore morphology of porous nanofibers prepared by SR pore−forming mechanism is not uniform, the pore size is generally 5−30 nm, and the porosity is more than 85% [65,94].

PVP and PEO are commonly used polymer materials and can be used as sacrificial components, which are removed by post−treatment (deionized water rinsing or high temperature extraction). We can control the proportion and distribution of pores by changing the sacrificial component content, but when the content exceeds 50%, the fibers removed will no longer be stable, so we must select the right proportion of polymer when preparing the precursor solution. For example, Yan et al. [95] used PAN and PVP as precursor materials for electrospinning, obtained porous carbon nanofibers by heat treatment to remove PVP, and in situ immobilized FeCo_2_S_4_ and MXene, which has high electrical conductivity and is a good capacitive material. MXene, which was first reported by Gogotsi’s team in 2011, has ultra−high electrical conductivity, a hydrophilic surface, and high chemical and mechanical stability [96,97]. Porous carbon nanofibers provide continuous channels for the rapid diffusion of electrolyte ions, a shortened transport path, and accelerated ion transport. The porous structure and surface modification endow the prepared carbon nanofibers with excellent electrical conductivity, remarkable ion transport capacity, and excellent electrochemical dynamic properties. The PVP content affects the pore capacity, size, and distribution density and the corresponding diameter, fracture strain, and other properties of the fibers after heat treatment.

Methaapanon et al. [98] prepared nanofibers with a core layer PAN/sheath layer ZnO−PVP by electrospinning, and the removal of PVP by high temperature treatment resulted in a porous structure and increased the specific surface area and the crystallinity of ZnO, which led to the enhanced activity of the photocatalytic degradation of methylene blue (MB). Ning et al. [99] prepared Ag nanoparticle−coated PEO−polyvinylidene fluoride (PVDF) nanofibers and removed the PEO using pure water to obtain porous PVDF nanofibers, and the specific surface area, the pore volume, and the pore size increased first and then decreased with the increase of the PEO molecular weight, which is related to the ratio of the PEO to PVDF content, which increased with the PEO content. These Ag−coated porous PVDF nanofibers have a good photocatalytic degradation activity for methyl orange (MO).

Porous structures can be obtained by the selective removal of the sacrificial phase in the post−treatment process, but the requirements for the components of the sacrificial phase and the post−treatment are strict.

## 3. Applications in Photocatalytic Degradation of Water Pollutants

Photocatalysis is a simple and efficient technology for photocatalysts to remove pollutants by stimulating its oxidation−reduction ability through light. The common photocatalytic particles include CdS [100,101], SnO_2_ [102,103], AgCl [104], and ZnO [105,106,107]. Single particles have limited applications due to their own light absorption, the compounding of electron−hole pairs, and lack of specific surface area. Researchers have formed co−catalytic composite systems by compounding two or more photocatalytic materials to compensate for their shortcomings and improve photocatalytic efficiency [108,109]. This system can be divided into type I heterojunctions, type II heterojunctions, type III heterojunctions, Z−scheme heterojunctions, and S−scheme heterojunctions [110,111,112,113,114,115]. The Z−scheme heterojunction is a compound of electrons from a semiconductor with a low CB and the holes from a semiconductor with a high VB, so that the electrons remain in the semiconductor with a high CB and the holes in the semiconductor with a low VB, promoting the spatial separation of electron−hole pairs [116]. In the S−scheme heterojunction, the CB of a semiconductor with a large negative CB position and the VB of a semiconductor with a large positive VB position retain photogenerated electrons and holes, respectively. Meanwhile, the meaningless photogenerated electrons and holes will be recombined. Therefore, the charge transfer path in this system is a macroscopic “step,” which makes the heterojunction highly redox capable [117]. The type I, II, and III heterojunctions are different from the Z−scheme and S−scheme heterojunctions in that they all belong to the electron leap to CB of another semiconductor and the hole leap to VB of another semiconductor, rather than the electron−hole complex. For instance, in the type II heterojunction formed by ZnO−In_2_O_3_, electrons leap from the VB side of ZnO to the VB side of In_2_O_3_, and holes leap from the CB side of In_2_O_3_ to the CB side of ZnO, inhibiting the complexation of photogenerated electron−hole pairs [118].

Photocatalytic particles are inevitably lost during the catalytic process, which affects the catalytic performance in the cycle, and sufficient photocatalyst must be added to ensure the catalytic performance, thus increasing the cost. By loading photocatalytic particles onto nanofiber membranes through electrospinning, the loss of catalysts during recycling can be greatly avoided, saving costs and increasing the recycling capacity. Table 1 shows the composition, heterojunction types, and photocatalytic efficiency of several common photocatalytic particle−laden nanofiber membranes.

### 3.1. Organic Pollutants

Organic pollutants are natural organic substances in the form of carbohydrates, proteins, fats, and other biodegradable synthetic organic substances as constituent pollutants. Organic pollutants widely exist in water, air, and soil, and only a few can spontaneously degrade. However, the degradation rate of organic pollutants is very slow. This is because they generally have macromolecular chains or benzene rings, which inhibit the growth of organisms and are resistant to light, chemical and biodegradation, so they are difficult to spontaneously degrade in nature. The photocatalytic degradation of organic pollutants is a green and clean technology, which has great application prospects.

#### 3.1.1. Organic Dyes

**Figure 7:**
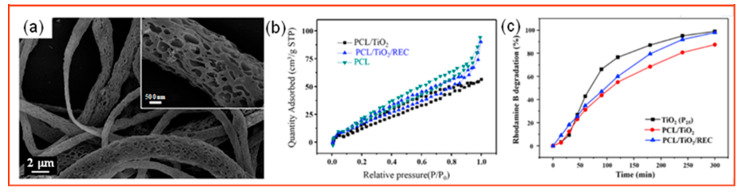
(**a**) SEM images of PCL/TiO_2_/REC fibers; (**b**) N_2_ adsorption−desorption isotherms of each sample; (**c**) degradation rate of each sample to rhodamine B [137].

The organic dye compounds in general industrial wastewater are not only very stable under normal conditions but also highly toxic, mutagenic, and carcinogenic to any organisms. Therefore, these toxic organic dyes must be effectively removed from industrial wastewater to prevent them from harming the ecosystem [129]. Porous fiber membranes loaded with photocatalytic particles were prepared by electrospinning for organic dye removal, which can effectively solve the hazards of organic dyes. The molecular formula of MB is C_16_H_18_ClN_3_S, and its solubility in water is very large. This is because it contains benzene ring, cyano group and methyl group, its structure is relatively stable, and it contains sulfur ions, which can inhibit the growth of bacteria, and its spontaneous degradation is extremely slow [130,131]. MB not only causes changes in water quality, but also causes irreversible damage when it accumulates too much in living organisms. Wu et al. [132] prepared a porous fiber membrane with pan as the skeleton and loaded it with TiO_2_ nanoparticles by electrospinning. TiO_2_, as a common photocatalytic material, has been widely used because of its advantages of low cost, good stability, and low toxicity [133,134]. TiO_2_ has the highest photocatalytic activity. By adding water−soluble polymer PVP and backbone polymer PAN to the electrospinning precursor and removing PVP by washing the fiber membrane with deionized water, a nano−PAN fiber membrane with high porosity on the surface can be obtained, exposing more TiO_2_ to the fiber surface, which not only increases the accessibility of TiO_2_ to light and generates more electrons and holes but also increases the contact between the TiO_2_ and the MB and the reaction active sites, thus improving the photocatalytic efficiency. The prepared pore size of 5–40 nm was analyzed by BJH. The pore size not only increased the adsorption of organic dyes but also immobilized the TiO_2_ particles to a great extent and prevented the shedding of catalysts in the photocatalytic process. The successful preparation of this porous fiber membrane loaded with photocatalytic particles provides a new direction for the design and development of a promising environmental application with adsorption−photocatalysis. The molecular formula of RhB is C_28_H_31_ClN_2_O_3_. It has more benzene rings, carboxyl groups and methyl groups than MB, which means that its structure is very stable and will not degrade spontaneously. Long−term exposure will bring huge harm to human health [135,136]. Gu et al. [119] prepared mesoporous ferroelectric Bi_6_Fe_2_Ti_3_O_18_ (BFTO) nanofibers with diameters of 100–150 nm by electrostatic spinning and post−treatment calcination. BFTO has a certain absorption intensity in visible light due to its lamellar structure with a proper band gap (2.5 eV). To improve the photocatalytic performance of BFTO, it was uniformly dispersed onto the fiber membrane by combining BFTO with electrostatic spinning. Through the subsequent calcination, many pores appeared on the fiber surface to increase the contact area of the BFTO with the pollutants. Au nanoparticles were also loaded onto the mesoporous BFTO fiber membrane by a simple photodeposition method because the plasma effect on the surface of Au nanoparticles improves the absorption of visible light by the BFTO nanofibers, and Schottky defects are generated at the Au and BFTO interface to promote the separation of electron–hole pairs, thus significantly improving the photocatalytic efficiency. To further investigate the effect of the iron polarization of BFTO nanofibers, a series of corona polarization experiments were conducted, and the structures proved that the photocatalytic efficiency of RhB was significantly improved after corona polarization. Therefore, porous Au/BFTO nanofiber membranes subjected to corona polarization have a wide range of application prospects for future industrial wastewater treatment. As shown in Figure 7, Tu et al. [137] selected a PCL with strong mechanical properties as the carrier and prepared PCL/TiO_2_ porous fiber membranes by electrospinning. Although TiO_2_ possesses the advantages of low price and good stability, it has poor reusability and may cause secondary pollution. Therefore, porous PCL fiber membranes with a large specific surface area were loaded with TiO_2_ to enhance the capacity and dispersion of TiO_2_ and achieve photocatalytic efficiency.

#### 3.1.2. Phenolic Pollutants

**Figure 8:**
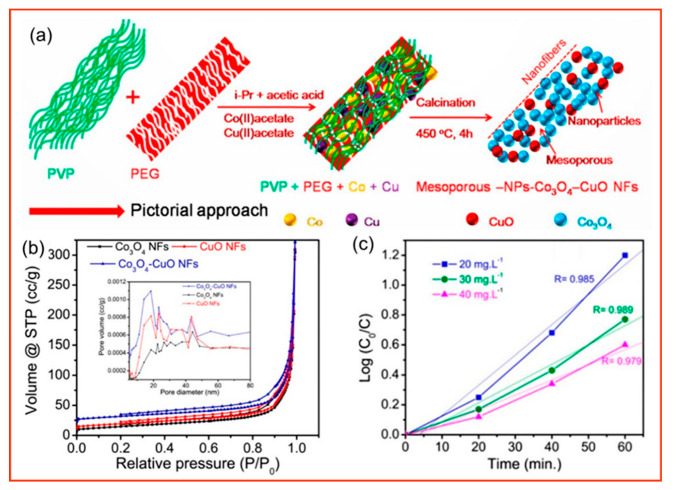
(**a**) Diagram of the formation mechanism of mesoporous composite Co_3_O_4_−CuO−NFs; (**b**) N_2_ adsorption isotherms and pore sizes of mesoporous Co_3_O_4_, CuO and composite Co_3_O_4_−CuO NFs (inset); (**c**) kinetics of degradation of 20, 30, 40 mg·L^−1^ of 4−nitrophenol (4−NP) by mesoporous composite Co_3_O_4_−CuO NFs [140].

Phenolic compounds are also an important class of wastewater pollutants produced by the chemical, paint, pesticide, biotechnology, and textile industries that can invade the human body through the digestive tract, the respiratory tract, and the skin and bind to proteins in the cellular protoplasm to cause cell inactivation. Nitrophenol is an important organic pollutant with an anti−degradation ability, carcinogenic substances, and mutagenic agents. The molecular formula of nitrophenol is C_6_H_5_NO_3_, and it has functional groups with stable structures such as benzene ring, nitro group, and hydroxyl group, so it is difficult to degrade spontaneously [138,139]. Once nitrophenol enters the human body, it is difficult to degrade and eliminate, making it very harmful to humans and a threaten to all aquatic organisms. As shown in Figure 8, Pradhan et al. [140] prepared nanofibers loaded with Co_3_O_4_ and CuO by electrostatic spinning using PVP and PEG as polymer templates. By removing the polymers through calcination, nanofibers with mesoporous structures can be constructed, thus increasing the specific surface area of the photocatalyst and the reactive sites, while mesopores shorten the electron transport path and increase the reactivity. The surface of the nanofibers prepared by electrostatic spinning contains many hydroxyl groups (−OH), which can improve the photocatalytic efficiency of p−nitrophenol and phenol.

**Figure 9:**
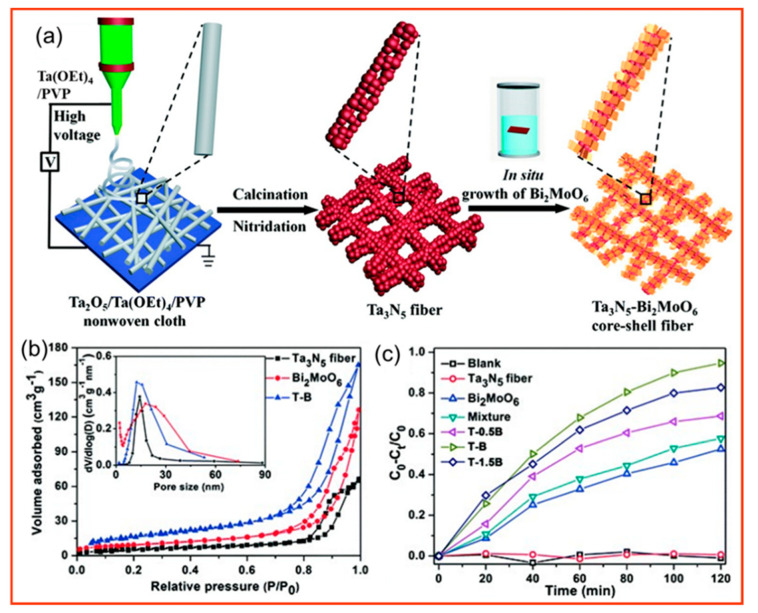
(**a**) Schematic of heterojunction Ta_3_N_5_/Bi_2_MoO_6_ nanofiber preparation; (**b**) N_2_ adsorption−desorption isotherms and pore size distribution of pure Ta_3_N_5_, Bi_2_MoO_6_ and T−B (inset); (**c**) degradation efficiency of 15 mg of various photocatalysts for 4−para−chlorophenol (4−CP) under visible light [142].

As shown in Figure 9, p−chlorophenol, which is also known as p−chlorophenol, is a toxic organic pollutant with strong irritating effects on the eyes, the skin, mucous membranes, and respiration. In animal experiments, p−chlorophenol can cause restlessness, accelerated breathing, and the rapid development of symptoms, such as weakness, tremors, clonic convulsions, coma, and even death, after a few minutes of ingestion. The molecular formula of p−chlorophenol is C_6_H_5_ClO. Because it has a benzene ring and contains chloride ions, it can inhibit bacterial growth and is not easy to spontaneously degrade [141]. Li et al. [142] prepared porous Ta_3_N_5_ nanofibers by electrostatic spinning−calcined nitridation and deposited Bi_2_MoO_6_ on their surface by in situ growth to form heterojunctions with Ta_3_N_5_, which inhibited the complexation of photogenerated electron–hole pairs and improved the photocatalytic activity for RhB and p−chlorophenol.

#### 3.1.3. Antibiotic

**Figure 10:**
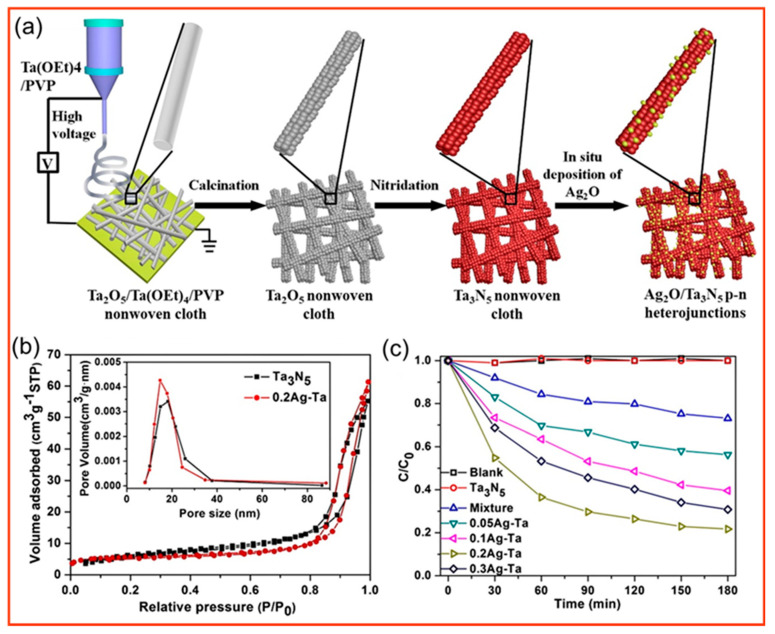
(**a**) Schematic of the synthesis of p−n heterojunction Ag_2_O/Ta_3_N_5_ nanofibers; (**b**) N_2_ adsorption−desorption isotherms and pore size distribution of pure Ta_3_N_5_ and 0.2Ag−Ta (inset); (**c**) degradation efficiency of TC (10 mg·L^−1^, 80 mL) during degradation with different catalysts (60 mg) [147].

Since the introduction of antipenicillin into medical therapy in 1942, hundreds of other antibiotics have been isolated or synthesized for the treatment of human and animal infections [143]. However, only approximately 30% of the antibiotic is absorbed and metabolized by the body, and a significant portion is excreted in the urine and feces in an unchanged active form [144], causing long−term pollution of the environment. The molecular formula of tetracycline (TC) is C_22_H_24_N_2_O_8_, and it has many structurally stable functional groups such as benzene ring and carbonyl group, which are difficult to degrade spontaneously [145,146]. TC is widely used for its effective treatment of trachoma, pneumonia, and acute diarrhea. The incomplete degradability of TC and the great toxicity of its intermediate products that result from its degradation have gradually intensified the pollution of the environment by TC. Meanwhile, the residual TC in the environment can also induce the resistance of microorganisms, resulting in the aggregation of drug−resistant colonies and the generation of drug−resistant genes, causing irreversible damage to the ecological environment and human health. As shown in Figure 10, Li et al. [147] prepared Ta_3_N_5_ porous nanofibers by electrostatic spinning−calcined nitridation and deposited Ag_2_O on their surface by in situ growth, forming p−n type heterojunctions, which are favorable for the separation and jumping of photogenerated electrons and holes and can mineralize organic pollutants and exhibit excellent stability, showing great application potential in TC contamination treatment in the environment.

**Figure 11:**
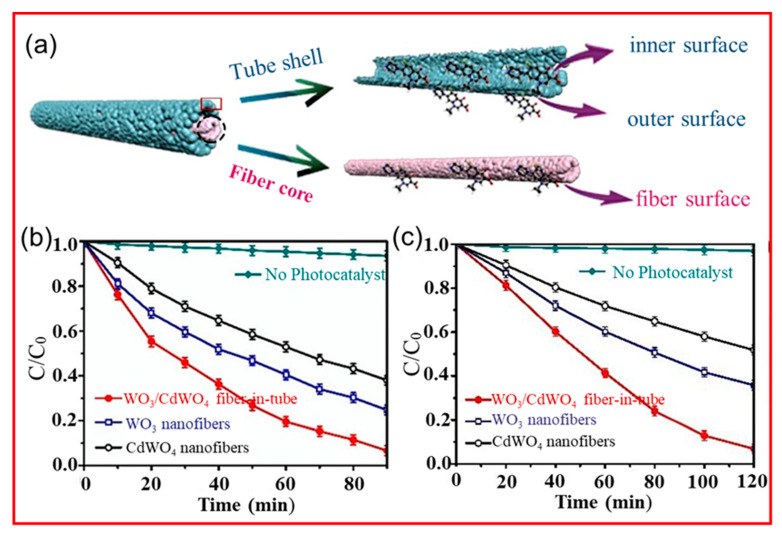
(**a**) Illustration of layered porous WO_3_/CdWO_4_ FITNs showing the three surfaces of CIP adsorption and catalytic sites; photocatalytic degradation efficiency of WO_3_, CdWO_4_ and WO_3_/CdWO_4_ FITNs for (**b**) CIP and (**c**) TC at room temperature [152].

Ciprofloxacin (CIP) is a widely used fluoroquinolone antibiotic [148]. Its molecular formula is C_17_H_18_FN_3_O_3_. It has structurally stable groups such as benzene ring, cyclopropyl group, carbonyl group, and contains fluorine atoms. It can inhibit bacterial growth and is difficult to spontaneously degrade [149,150]. Due to its high solubility, bacterial resistance, and difficulty in degradation, its long−term bioaccumulation can have genotoxic effects on aquatic organisms and humans [151]. As shown in Figure 11, Rong et al. [152] prepared fibers in tubes loaded with Z−scheme heterojunction WO_3_/CdWO_4_ graded porous structures (the inner and outer surfaces of the sheath layer and the outer surface of the core layer have porous structures) by electrostatic spinning. Given that WO_3_ and CdWO_4_ have different band gap properties, the formed Z−scheme heterojunction can increase the total wavelength of light absorption, make photogenerated electron holes migrate to the photocatalyst with the lowest CB/highest VB, induce the separation of electron holes, inhibit their compounding, and increase the substances that can participate in redox reactions, thus improving the photocatalytic efficiency of TC and CIP. Such nanofibers in tubes containing both Z−scheme heterojunctions and special graded porosity have more void fraction, larger specific surface area, more reactive active sites, and shorter ion transport paths, which can maximize the efficiency of each component and achieve high photocatalytic efficiency.

### 3.2. Inorganic Pollutants

**Figure 12:**
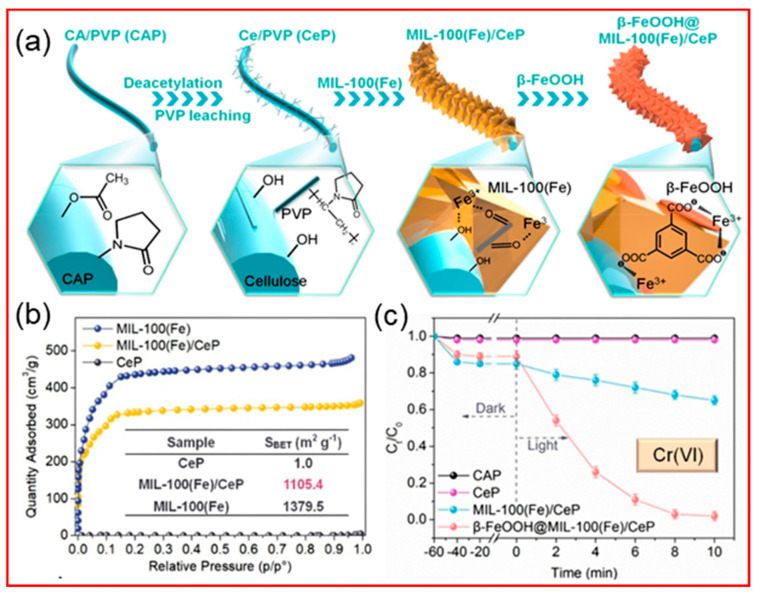
(**a**) Schematic of the preparation of porous core sheath β−FeOOH@MIL−100(Fe)/CeP NFM; (**b**) N_2_ adsorption isotherms of MIL−100(Fe) and MIL−100(Fe)/CeP NFM and pore structure parameters (inset); (**c**) In the presence of CeP, CAP, and under visible light irradiation, the MIL−100(Fe) and MIL−100(Fe)/CeP NFM photocatalytic activity curves for Cr(VI) in the presence of CeP, CAP [156].

The inorganic pollutants in water are continuously increasing with the rapid development of modern industry. These pollutants affect the pH in water, inhibit the growth of microorganisms, and hinder the self−purification of water. Among many inorganic pollutants, hexavalent Cr(VI) is an extremely dangerous heavy metal ion [153]. Cr(VI) mainly comes from metal electroplating, tanning, mining, printing, photography, and other industries [154]. Given its non−biodegradability, excessive toxicity, and carcinogenicity, which can cause liver damage and lung cancer, the effective removal of Cr(VI) from water bodies or the reduction of Cr(VI) to the less toxic Cr(III) has attracted great attention [155]. As shown in Figure 12, Lu et al. [156] prepared nanofibers with a porous surface by adding hydrophilic PVP to the electrospun polymer precursor and then synthetically grew β−hydroxy iron oxide modified metal organic skeleton MIL−100(Fe) (β−FeOOH@MIL−100(Fe)) photocatalysts in situ on its surface, which resulted in ultra−high MIL−100(Fe) loading through coordination and through good MB and Cr(VI) photocatalytic degradation due to the good dispersion of β−FeOOH nanorods.

### 3.3. Bacteria

**Figure 13:**
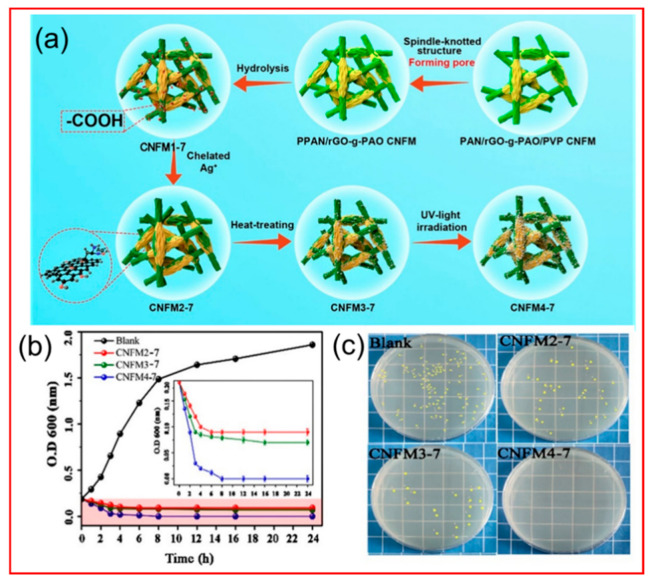
(**a**) Schematic of the preparation of P−PAN/rGO−g−PAO@Ag+/Ag CNFM; (**b**) growth of E. coli on CNFMs; inset: magnified view of the red area; (**c**) incubation in solid medium containing CNFM2−7, CNFM3−7 and CNFM4−7 and blank at 37±1°c for 24h to observe E. coli colony formation situation [162].

Sewage often contains pathogens that are harmful to human and animal health [157], porous materials play an important role in antibacterial [158]. Escherichia coli is the most common bacterial contaminant, and is composed of cell wall, cell membrane, cytoplasm, nucleus, ribosome, etc. It is an important indicator for detecting bacteria in water bodies that can transmit disease through water [159]. After invading the human body, E. coli can cause physical diseases under certain conditions, such as cholecystitis, cystitis, and diarrhea, which may affect life in serious cases. The ·O^2−^ and ·OH produced in the photocatalytic process can decompose organic substances such as proteins on the cell membrane of E. coli, and destroy the cell membrane. Because E. coli lacks the isolation and protection of the cell membrane, active substances can invade the cells, resulting in inactivation of E. coli until death [160,161]. As shown in Figure 13, Han et al. [162] prepared porous PAN/reduced graphene oxide (rGO)−polyamide oxime (PAO) (P−PAN/rGO−g−PAO@Ag+/Ag) nanofiber membranes loaded with Ag+ by electrostatic spinning using PVP as the porogenic agent. PAN has good thermal and chemical stability, corrosion resistance, and high mechanical strength, while PVP has good film formation and water solubility and features the easy removal of pore formation. Ag+ can grow on the fiber surface and be reduced to Ag by PAO adsorption, making it recyclable, and the synergistic effect of two Schottky structures, rGO/Ag and Ag+/Ag, on the fiber membrane shows good photocatalytic activity against bacterial microorganisms. The photocatalytic degradation rate constant of CNFM4−7 for the dye benzoic acid (BA) is approximately several to ten−times higher than that of CNFM2−7 and CNFM3−7, and the inactivation rate of E. coli is up to 100% due to the presence of rGO−g−PAO@Ag+/Ag Schottky junctions, which can cause surface plasmon resonance and enhance the photocatalytic activity.

## 4. Relationships between Porous Structure and the Properties of Nanofibers

Unlike the BF pore−forming mechanism, which can only prepare nanofibers with porous surface, the nanofibers prepared by the VIPS, NIPS, TIPS and SR pore−forming mechanism have porous structures on the surface and inside of the fibers, which can reveal more photocatalytic particles, increase the contact area with organic pollutants, shorten the ion transport path, and show stronger adsorption capacity and photocatalytic efficiency. However, compared with the nanofibers with porous surface prepared by the BF pore−forming mechanism, they also show greater disadvantages, such as significantly reducing the mechanical properties of the fibers. As a result, the electrospun porous nanofibers are easy to fracture in the process of degrading the pollutants in water. This disadvantage may reduce the activity and reusability of photocatalyst [163], even worse, it may produce secondary pollution. Therefore, it is necessary to develop some new strategies to improve the mechanical properties of porous nanofibers.

The mechanical properties can be improved by preparing core−sheath nanofiber. Xu et al. [164] prepared core−sheath nanofibers with a porous sheath layer and a solid core layer by combining coaxial electrospinning and post−processing. The existence of a core−sheath structure effectively improves the mechanical properties of the nanofibers. Meanwhile, the porous structure of the sheath can increase the contact area and adsorption sites, thus ensuring the adsorption properties of nanofibers. You et al. [165] prepared core−sheath nanofibers with a porous TiO_2_ skeleton by a combination of self−assembly and electrospinning. The core layer of the porous TiO_2_ skeleton and sheath layer can significantly improve the mechanical properties of porous nanofiber films. Changing the distribution of pores can also be used to improve the mechanical properties of nanofibers.

Some nanofibers with special structures, such as beads−on−the−string nanofibers or Janus nanofibers, also have good mechanical properties. For example, Wang et al. [166] prepared porous bead−on−string poly(lactic acid) fibrous membranes, in which the pores were mainly distributed in beads, for air filtration. Wang et al. [167] prepared electrospun nanofibers with a Janus structure, with pores mainly distributed on one side of the nanofibers. This method can minimize the loss of mechanical properties while ensuring the existence of the porous structure. Similarly, many multiple−chamber nanofibers, which are produced using multiple−fluid electrospinning and have been reviewed in some articles [168,169,170,171,172], can be similarly explored for achieving a general fine mechanical performance with the support from one of the chambers.

The mechanical properties of electrospun nanofibers can also be improved by coating the cast film on the electrospun nanofiber film. For example, Liu et al. [173] combined electrospinning with cast membranes, and made comprehensive use of different fiber films, which not only improved the overall mechanical properties, but also enabled the drug to be released slowly and stably through porous structure in the second release stage, so as to achieve biphasic controlled release.

## 5. Conclusions and Perspectives

In recent years, electrospinning has become a common method of preparing nanofibers with various structures due to its advantages of simple operation and controllability, and nanofibers with various structures can be prepared by changing the number of polymer fluids, the type of electrospinning head, and the working parameters. This paper reviews the formation mechanism of porous fiber membranes prepared by electrospinning: BF, VIPS, NIPS, TIPS, SR. The porous structure can increase the specific surface area of nanofibers and the area in contact with the light source while shortening the electron transport path and increasing the reaction activity, thus showing a wide range of application prospects in photocatalysis. From the perspective of the environment and the economy, loading photocatalytic particles on porous nanofibers can not only effectively remove organic pollutants, heavy metal ions, and harmful bacteria in water and reduce water pollution but also greatly avoid the loss of photocatalysts in recycling and save costs.

To improve the application prospects of porous nanofibers loaded with photocatalysts in the catalytic degradation of pollutants in water, the following aspects should be emphasized: (1) more effective methods are needed to construct regular pore structures; (2) the presence of many porous structures on fibers will inevitably have an impact on the mechanical properties of fibers and affect the recyclability of fibers, and the study of a highly effective method for increasing the mechanical properties of fibers will widen the range of applications; and (3) the effective and stable large−scale preparation of porous nanofibers loaded with photocatalysts is also a major challenge in industrial applications at present. With the in−depth research on photocatalysts and electrostatic spinning, these difficulties can be overcome in the near future in order to solve various environmental problems and achieve a sustainable environment.

## Figures and Tables

**Table 1:** Application of nanofibers loaded with photocatalytic particles for photocatalytic degradation of pollutants in water.

Fiber Membrane Composition		Types of Heterojunctions	Photocatalytic Reactants	Photocatalytic Efficiency		References
Fiber Skeleton	Photocatalytic Particles			Conversion Rate	Repeatability	
Bi_6_Fe_2_Ti_3_O_18_ (BFTO)	Au@ (BFTO)	−	RhB	90%	≥4 run	[119]
Polyethersulfones (PES)	(MWCNTs)−Fe_3_O_4_	−	Cr(VI)	99%	≥5 run	[120]
Chitosan	g−C_3_N_4_/TiO_2_	−	Cr(VI)	90%	≥5 run	[121]
PVDF/PAN	CuO−ZnO	type I	MO	90%	≥5 run	[122]
BaTiO_3_–TiO_2_	BaTiO_3_–TiO_2_	type II	RhB	99.8%	≥5 run	[123]
PAN	CNT/TiO_2_−NH_2_	type II	Phenol	99.7%	−−	[124]
g−C_3_N_4_/Nb_2_O_5_	g−C_3_N_4_/Nb_2_O_5_	−	RhB, phenol	RhB: 98.1%, Phenol: 100%	≥4 run	[125]
In_2_TiO	In_2_O_3_/In_2_TiO	type II	RhB, Lvofloxacin (LEV)	RhB: 97.1%, LEV: 86%	≥5 run	[126]
g−C_3_N_4_	WO_3_/g−C_3_N_4_	Z−scheme	Phenol, Cr(VI)	Phenol: 75%, Cr(VI): 90%	≥3 run	[127]
TiO_2_	Bi_2_O_3_/TiO_2_	S−scheme	Phenol	Phenol: 50%	−	[128]

## Data Availability

The figures referenced in this review are all licensed.
